# The Treatment of Cervical Adenitis

**Published:** 1899-08-26

**Authors:** 


### THE TREATMENT OF CERVICAL ADENITIS.

In the treatment of the adenitis of children, which in a very large proportion of cases is tuberculous, and often leads to suppuration and ugly searing of the neck, there are several points worthy of attention, which were well laid down by Mr. George Morgan, of Brighton, when speaking at the Portsmouth meeting. It is necessary to inform oneself carefully as to the cutaneous, and more especially the mucous surfaces, which are drained by the particular set of glands affected in each case. It is by way of the teeth and gums, the tonsils, and the naso-pharynx, especially the vault when affected by adenoids, that the tubercle bacillus gains admission; and the tonsils and the naso-pharynx are the chief points of entering in those cases in which the deep cervical glands about the angle of the jaw are affected, even though in the case of the tonsil there be no manifest enlargement. In regard to treatment much may be done without operative interference when the glands are discovered in an early stage. Tonics, cod liver oil, fresh air, sunshine, and sea air if possible, are a matter of course, and teeth, tonsils, naso-pharynx, stomatitis, and small ulcers of the lips and gums, should all be promptly treated. Even in the application of remedies it is important to bear in mind the area from which the lymphatics have their source, for by applying remedies to this area they very effectually influence the glands in question. For superficial glands draining the skin iodide of potassium ointment well rubbed in is of service, but in cases in which the glands of the deep cervical group are affected an application composed of iodine 12 grains, potass. iodid. 15 grains, ol. menth. pip. minim. 2, glycerin. 1 fluid ounce, may be painted on the tonsils, and, although it will not answer in all cases, yet if applied before softening takes place "it undoubtedly reduces the majority." When operation is required the proceedings differ according as the gland is completely broken down or not. If it is broken down it is better to incise the capsule at once, scrape it out with a sharp spoon, and then dress the inside with pure carbolic acid. If, after this, the capsule can be teased out, so much the better, but it is a pity to waste time in trying to dissect out the remains of a gland which is in reality only a bag of pus. It is sure to burst and infect the wound. Another point is that in excising glands no pressure should be made on them, either by the fingers or forceps, and that if several glands are affected the lower one should be taken first, working up in the opposite direction to the lymph current. If the glands are squeezed their tuberculous contents are apt to be forced onwards; thus fresh glands become implicated, and a recurrence of the disease is apt to show itself very shortly after the operation, which is very disappointing. As a practical point, it is to be added that where there are caseous glands and adenoids, unless the glands are mere bags of pus, it is a good rule to excise the adenoids first and deal with the glands afterwards.

